# Parental perceptions, information sources, and practices related to childhood immunization and HPV vaccine awareness in Saudi Arabia: an observational study

**DOI:** 10.3389/fpubh.2026.1992535

**Published:** 2026-10-22

**Authors:** Ayesha Yasmeen, Mamoon H. Syed, Amani Khardali, Moaddey Alfarhan, Hilal A. Thaibah, Hadi A. Almansour, Saeed A. Al-Qahtani, Yasir M. Khawaji, Sultan A. Qadiri, Abdulelah I. Faqihi, Jaber D. Hallush, Ali H. Alhazmi, Thamir M. Alshammari

**Affiliations:** 1 Department of Pharmacy Practice, College of Pharmacy, Jazan University, Jazan, Saudi Arabia; 2 Pharmacy Practice Research Unit, College of Pharmacy, Jazan University, Jazan, Saudi Arabia; 3 College of Pharmacy, Jazan University, Jazan, Saudi Arabia

**Keywords:** children, cross-sectional survey, immunization, parents, Saudi Arabia, vaccine

## Abstract

**Background:**

Parents’ perceptions, information sources, and household vaccination practices influence routine childhood immunization and may also shape responses to newer vaccines. We conducted an updated Saudi survey using an adapted instrument and included a human papillomavirus (HPV) vaccine module to assess self-reported awareness and recommendation intention.

**Methods:**

A cross-sectional online survey was conducted among parents in Saudi Arabia from June to July 2025. The Arabic questionnaire covered vaccination awareness and perceptions, information sources, parent-reported practices and experiences, with two HPV-specific items. Descriptive analyses used chi-square/Fisher’s exact tests. Multivariable binary logistic regression examined factors associated with HPV vaccine awareness, and an exploratory multinomial logistic regression examined HPV vaccine recommendation intention.

**Results:**

A total of 513 parents were included. Self-reported general vaccine awareness was 96.9%. Routine vaccination uptake was 98.8%, and 90.6% reported completing recommended vaccinations. Awareness of national immunization programs was 57.9%, and 61.4% reported satisfaction with vaccination information in the media; 71.7% did not believe vaccination is harmful. Health workers were the most frequently selected source of vaccination information (60.0%). HPV vaccine awareness was 68.6%, and 52.4% would recommend HPV vaccination for their child, while 19.1% selected “I do not know.” In adjusted analysis, HPV vaccine awareness was higher among females (aOR 1.87; 95% CI 1.23–2.86), among parents in all other regions compared with the Southern region (aORs 2.02–3.38), among those reporting problems during vaccination (aOR 2.82; 95% CI 1.76–4.51), and among those expressing either a harmful or non-harmful view of vaccination compared with “I do not know.” In the exploratory multinomial analysis, HPV vaccine awareness was associated with a lower relative likelihood of responding “I do not know” rather than “Yes” to HPV vaccine recommendation intention (aRRR 0.07; 95% CI 0.04–0.13).

**Conclusion:**

Routine childhood immunization was strongly established, while HPV awareness and recommendation intention were less uniform, with a sizable undecided group. Findings support stronger provider communication and credible public messaging, particularly for lower-awareness regions and uncertain parents.

## 1 Introduction

Vaccination remains one of the most effective public health interventions, preventing an estimated 3.5 to 5 million deaths each year worldwide (1). Yet high coverage alone does not always mean that parents are fully confident about vaccination. Sustained immunization also depends on trust, demand, and confidence, particularly when parents encounter questions about vaccine benefits, harms, or less familiar vaccines (2). At the household level, these decisions are shaped by the information sources parents trust and by their previous experiences with routine childhood immunization.

In Saudi Arabia, the earlier Hail survey found generally supportive parental views toward routine childhood immunization, but it also showed that acceptance and recommendation were linked to parents’ information sources and vaccination experiences (3). Since then, parents have been making vaccination decisions in a more crowded information environment, where advice from health professionals sits alongside social media content and wider public narratives (4, 5). A follow-up survey conducted in 2025 using an adapted version of the earlier instrument in a multi-region online sample therefore helps identify which patterns remain consistent and where parental views may differ.

Vaccination-related awareness, knowledge, confidence, hesitancy, acceptance, and recommendation intention are related but distinct concepts. Vaccine confidence concerns trust in vaccines and vaccination systems, whereas vaccine hesitancy refers to delay in acceptance or refusal of vaccination despite the availability of vaccination services and is influenced by several factors, including confidence (6, 7). Saudi studies have also demonstrated variation in parental awareness and attitudes toward childhood immunization despite high routine vaccination uptake (8). In the present study, awareness refers specifically to self-reported familiarity based on responses to individual questionnaire items, whereas recommendation intention refers to the participant’s stated willingness to recommend vaccination. These measures should not be interpreted as direct assessments of vaccine knowledge, confidence, hesitancy, or acceptance.

We also included an HPV vaccine module because adolescent-targeted vaccines may reveal gaps that are less visible when parents are asked only about routine childhood immunization. Human papillomavirus (HPV) vaccination is central to cervical cancer prevention, and the World Health Organization places it alongside screening and treatment in the global strategy for cervical cancer elimination (9). The WHO position paper also supports the effectiveness and safety of HPV vaccines and provides guidance on flexible program delivery, including simplified dosing schedules in appropriate settings (10). By late 2021, Saudi Arabia had included the HPV vaccine in its national vaccination programme, with rollout among girls aged 9–13 years planned for the first quarter of 2022 (11). Current Ministry of Health guidance lists male and female adolescents aged 11 or 12 years, as well as previously unvaccinated individuals through age 26 years, as eligible for HPV vaccination (12).

Recent Saudi studies indicate that parental awareness, acceptance, and hesitancy regarding HPV vaccination remain variable. Among 551 parents of females aged 9–26 years surveyed in Saudi Arabia, 61.5% were aware of the HPV vaccine and 68.4% intended to vaccinate their daughters (13). In a Riyadh study, 41.3% of parents were aware of the HPV vaccine, while 60% believed that their daughters should receive it (14). More recently, a study of 667 parents of adolescent females across Saudi Arabia using a 9-item modified Vaccine Hesitancy Scale found that 34% were hesitant about HPV vaccination (15). National coverage estimates indicate that, by 2023, 39.0% of girls turning 15 years in Saudi Arabia had received two doses of the HPV vaccine (16). Reported barriers among Saudi parents have included limited awareness and insufficient information, fear of vaccines or needles, conflicting medical advice, lack of healthcare-provider recommendation, and practical inconvenience related to vaccination (13, 15). Together, these findings indicate that HPV vaccine awareness, acceptance, hesitancy, and uptake remain uneven across Saudi populations. However, previous studies have predominantly examined HPV-specific knowledge, attitudes, acceptance, or hesitancy, often within selected populations or regions. Less is known about HPV vaccine awareness and recommendation intention in the context of parents’ broader perceptions, information sources, and reported practices related to routine childhood immunization across multiple Saudi regions. Assessing HPV vaccine awareness and intention to recommend it within the same survey therefore allowed us to examine whether the high reported uptake of routine childhood immunization was accompanied by similarly high awareness of, and willingness to recommend, HPV vaccination.

This cross-sectional survey assessed vaccination awareness, perceptions, information sources, and parent-reported vaccination practices among Saudi parents using an instrument adapted from a previously published Saudi study. The survey also included an HPV vaccine module to assess awareness of the HPV vaccine and parents’ intention to recommend it for their child. In addition, we examined sociodemographic and perception-related factors associated with HPV vaccine awareness to identify where awareness was most uneven across groups.

## 2 Methods

### 2.1 Study design and setting

We conducted a cross-sectional online survey of parents residing in Saudi Arabia. The questionnaire was administered via Google Forms and distributed through social media and the investigators’ networks to recruit respondents across regions. Data were collected from 13 June 2025 to 3 July 2025. The final analytic sample included 513 eligible respondents.

### 2.2 Participants and recruitment

Participants were eligible if they were aged 18 years or older, were parents, resided in Saudi Arabia, and provided electronic informed consent. Eligibility was not restricted according to the age of their children. The survey link was shared through WhatsApp and X (formerly Twitter), as well as directly through the investigators’ contacts and professional networks. No paid or targeted advertising was used. Participation was voluntary. Before accessing the questionnaire, participants viewed a brief study description and an electronic consent statement, and could proceed only after indicating consent. Participants were also informed that the survey required approximately 10 min to complete.

### 2.3 Sample size

The study used a non-probability convenience sampling approach. An initial recruitment target of approximately 385 respondents was estimated using the Raosoft online calculator, assuming a 50% response distribution, 95% confidence level, and 5% margin of error. Because recruitment was not probability based, these parameters were used only to provide an approximate planning target and should not be interpreted as establishing a ± 5% population margin of error. Recruitment continued beyond this target to increase the number of observations available for subgroup and multivariable analyses. For the primary binary logistic regression, the less frequent outcome category comprised 161 participants, and the model included 15 regression parameters, corresponding to approximately 10.7 observations in the less frequent outcome category per parameter.

### 2.4 Survey instrument and measures

The survey instrument was adapted from a previously published Saudi study on parental perceptions and acceptance of childhood immunization (3). Core items addressing vaccination awareness and perceptions, information sources, and parent-reported vaccination practices were retained or adapted from the earlier instrument, while demographic response categories were modified for the present multi-region sample. Region was added as a demographic variable, and education, occupation, and household income categories were expanded or refined. Two newly developed HPV-specific items assessing HPV vaccine awareness and intention to recommend HPV vaccination were also added as a focused module. The questionnaire covered four domains: participant characteristics, vaccination awareness and perceptions, vaccination information sources, and parent-reported vaccination practices and experiences. The English source questionnaire was translated into Arabic by the student co-authors and subsequently reviewed by six bilingual study authors, who reached agreement on the translated wording. The Arabic version was then independently reviewed by two faculty members who were not part of the study team and had extensive experience in survey research. Their suggestions related to language, grammar, and terminology were incorporated following agreement among the study authors. The revised Arabic questionnaire was then pilot-tested with 20 parents to assess clarity and comprehensibility, wording, response options, survey flow, and completion time. No reliability testing, psychometric analysis, or formal quantitative content-validity assessment was performed in the present study. Following pilot testing, demographic response categories were refined, regional categories were consolidated, and the recommended-vaccination completion item was clarified for parents of children younger than 5 years. The 20 pilot participants were not included in the final analytic sample. The resulting final Arabic version was used for data collection, and an English version was prepared for reporting.

Participant characteristics included age group, gender, region, education, employment sector, household income, and number of children aged <5 years.

Vaccination awareness and perceptions were assessed using individual questionnaire items with Yes/No/I do not know response options. Items addressed self-reported awareness of vaccination, perceived harmfulness of vaccination, awareness of the national immunization program, receipt and maintenance of an immunization certificate, satisfaction with vaccination information in the media, and willingness to recommend vaccination. HPV vaccine awareness was assessed using a single Yes/No item, while intention to recommend HPV vaccination was recorded as Yes/No/I do not know. The two HPV-specific items were not derived from a validated HPV-specific scale; they underwent the bilingual author review, independent expert review, and pilot assessment for clarity and comprehensibility described above. All awareness, perception, and recommendation items were analyzed individually; no composite or validated scale of vaccine knowledge, confidence, hesitancy, or acceptance was administered. Accordingly, “awareness” in this study refers to self-reported awareness based on responses to the relevant questionnaire item, while “recommendation intention” refers specifically to the participant’s stated willingness to recommend vaccination. “I do not know” response options were included for relevant perception items to capture indecision rather than forcing a Yes/No choice. Items were sequenced from general vaccination questions to the HPV module (awareness and intention), followed by parent-reported practices and experiences.

Vaccination purposes and information sources were assessed using multi-select items. Each listed option was analyzed separately according to whether it was selected, regardless of other options selected by the same respondent. Responses indicating “All of the above” were counted as selection of each individual option included in that item. Frequencies and percentages were therefore reported for each individual purpose and information source, and percentages could exceed 100% because respondents could select more than one response.

Parent-reported practices and experiences included whether the participant had vaccinated their children, whether their child had experienced any problems during vaccination, whether their child had experienced any problems after vaccination, whether their child had experienced a parent-reported side effect following vaccination that resulted in a doctor or hospital visit, and completion of recommended vaccinations up to age 5 years or to the child’s current age if younger than 5 years. The questionnaire did not provide a predefined clinical definition or severity threshold for a “problem” or “side effect,” and these responses were not clinically verified. The doctor/hospital-visit item was administered as a separate question to all respondents and was not conditional on an affirmative response to the preceding post-vaccination-problem item.

An English-language version of the complete questionnaire is provided as Supplementary Material.

### 2.5 Data collection

The questionnaire was administered using Google Forms. A total of 630 responses were received. Of these, 20 incomplete questionnaires and 97 responses from individuals who did not meet the eligibility criterion of being a parent were excluded, resulting in a final analytic sample of 513. Google account sign-in was required to enable the “Limit to 1 response” setting; however, email addresses were not collected by the study team. IP addresses and device identifiers were not collected or used to restrict participation. Potential duplicate submissions were additionally screened manually by comparing timestamps, demographic characteristics, and response patterns; no duplicate submissions were identified.

Post-vaccination problems and events leading to a doctor or hospital visit were treated as parent-reported experiences and were not interpreted as clinically confirmed outcomes.

### 2.6 Statistical analysis

All analyses were conducted using R statistical software version 4.5.0 and RStudio version 2025.05.0 + 496. Responses were screened for eligibility and for completeness of the variables required for analysis; ineligible or incomplete records were excluded. Categorical variables were summarized as frequencies and percentages. Bivariate associations were assessed using Pearson’s chi-square test or Fisher’s exact test when expected counts were small. All tests were two-sided and statistical significance set at *p* < 0.05. For bivariate analyses, effect estimates were reported alongside *p* values where appropriate. Because the general vaccine-awareness item had sparse counts in the “No” and “I do not know” categories, Cramer’s V with 95% confidence intervals, calculated using the noncentral chi-square method, was used as the omnibus effect-size measure for these comparisons. For the binary HPV vaccine-awareness outcome, crude odds ratios (ORs) with 95% confidence intervals were calculated for category-specific comparisons. No formal adjustment for multiple comparisons was prespecified; therefore, bivariate *p* values are unadjusted and should be interpreted as exploratory, with emphasis placed on effect estimates and confidence intervals rather than statistical significance alone.

For the multivariable analysis of HPV vaccine awareness, covariates were selected using a domain-based approach based on their conceptual relevance and the structure of the available data rather than on bivariate statistical significance. Binary logistic regression was performed with HPV vaccine awareness (Yes/No) as the dependent variable. The primary adjusted model included age group, gender, region, educational level, monthly household income, perceived harmfulness of vaccination, and parent-reported problems during vaccination. Educational level was grouped as high school or less, bachelor’s degree, and postgraduate education because of the small numbers of participants in the primary- and middle-school categories. Reference categories were 18–35 years for age, male for gender, Southern region for region, high school or less for education, monthly household income ≤SAR 5,000 for income, “I do not know” for perceived vaccine harmfulness, and No for problems during vaccination. Adjusted odds ratios (aORs) with 95% confidence intervals were reported.

Occupation was not included in the primary model because it comprised 12 heterogeneous categories, several with small cell counts, and inclusion of all categories would substantially increase the number of model parameters. Vaccination information sources were also not included as a single covariate because they were collected as non-mutually-exclusive multi-select responses. To assess the robustness of the primary model to these exclusions, a sensitivity analysis was performed in which occupation and four binary indicators representing reported use of media, health workers, friends or relatives, and the Ministry of Health mobile service as vaccination-information sources were additionally included.

Because HPV vaccine recommendation intention was recorded in three categories (Yes, No, and “I do not know”), an additional exploratory multinomial logistic regression was performed with recommendation intention as the dependent variable. “Yes” was used as the reference outcome category, while “No” and “I do not know” were retained as distinct categories. The model included age group, gender, region, educational level, monthly household income, perceived harmfulness of vaccination, and HPV vaccine awareness. The same grouped educational categories and reference categories for age, gender, region, education, income, and perceived vaccine harmfulness described above were used, with “No” as the reference category for HPV vaccine awareness. Adjusted relative risk ratios (aRRRs) with 95% confidence intervals were reported.

### 2.7 Ethical considerations

The study protocol and questionnaire were reviewed and approved by the Standing Committee for Scientific Research at Jazan Health Cluster, Saudi Arabia (No. 2562). The survey opened with an electronic consent statement outlining the study purpose, voluntary participation, and confidentiality. Participants could discontinue at any point before submitting the questionnaire. No direct identifiers (such as names, national identification numbers, or phone numbers) were collected. Data were exported from Google Forms and stored in password-protected files with access restricted to the study team. Results are reported in aggregate.

## 3 Results

### 3.1 Participant characteristics

A total of 513 parents were included in the analysis. The sample was evenly distributed by sex (50.1% male; 49.9% female). Most participants were aged 36–55 years (59.1%), followed by 18–35 years (25.5%) and 56 years or older (15.4%). Nearly two-thirds of participants had bachelor’s-level education (66.3%), and 18.5% reported postgraduate education. Responses were obtained from all regions, with the highest proportions from the Eastern region (25.7%) and Northern region (24.9%), followed by the Central (20.5%), Southern (14.6%), and Western (14.2%) regions. Nearly half of participants (46.9%) reported a monthly household income of SAR 10,001–20,000 (approximately US$2,667–5,333), followed by SAR 5,001–10,000 (approximately US$1,334–2,667; 37.4%). The largest occupational group worked in education/teaching (23.6%), followed by industry (11.9%) and tourism (11.5%). The largest proportion of participants (29.0%) reported having three children younger than 5 years; 25.1% reported one child and 24.0% reported two children. Additional characteristics are presented in Table 1.

**Table 1:** Sociodemographic characteristics of participating parents (*n* = 513).

Characteristic	*n* (%)
Gender	
Male	257 (50.1)
Female	256 (49.9)
Age (years)	
18–35	131 (25.5)
36–55	303 (59.1)
56 or older	79 (15.4)
Education level	
Primary school	2 (0.4)
Middle school	9 (1.8)
High school	67 (13.1)
Bachelor’s degree	340 (66.3)
Postgraduate	95 (18.5)
Region	
Central	105 (20.5)
Eastern	132 (25.7)
Northern	128 (24.9)
Southern	75 (14.6)
Western	73 (14.2)
Occupation sector	
Education (teaching)	121 (23.6)
Industry	61 (11.9)
Tourism	59 (11.5)
Unemployed	55 (10.7)
Health services	46 (9.0)
Agriculture	42 (8.2)
Financial services	41 (8.0)
Free trade	26 (5.1)
Legal	21 (4.1)
Military	18 (3.5)
Student	17 (3.3)
Retired	6 (1.2)
Number of children <5 years	
1	129 (25.1)
2	123 (24.0)
3	149 (29.0)
4	97 (18.9)
>4	15 (2.9)
Monthly household income (SAR)*	
≤5,000	39 (7.6)
5,001–10,000	192 (37.4)
10,001–20,000	241 (46.9)
≥20,000	41 (8.0)

*Approximate US dollar equivalents are based on SAR 3.75 = US$1.

### 3.2 Self-reported general vaccine awareness

Self-reported general vaccine awareness was high in the study sample, with 497/513 participants (96.9%) answering “Yes” to the item “Do you know about vaccines?” Associations between self-reported general vaccine awareness and age, gender, and region were small and not statistically significant (Cramer’s V = 0.059, 0.073, and 0.069, respectively; all *p* > 0.05). Parents reporting completed recommended vaccinations were more likely to answer “Yes” to the general vaccine-awareness item than those who did not, although the magnitude of association was small (Cramer’s V = 0.140; 95% CI: 0.041–0.221; Fisher’s exact *p* = 0.024) (Table 2).

**Table 2:** Self-reported general vaccine awareness by participant characteristics (*n* = 513).

Factor	Category	I do not know, *n* (%)	No, *n* (%)	Yes, *n* (%)	Total (*n*)	Cramer’s V (95% CI)	*p*-value
Age (years)	18–35	2 (1.5)	5 (3.8)	124 (94.7)	131	0.059 (0.000–0.101)	*p* = 0.504
	36–55	3 (1.0)	5 (1.7)	295 (97.4)	303		
	56 or older	0 (0.0)	1 (1.3)	78 (98.7)	79		
Gender	Female	4 (1.6)	7 (2.7)	245 (95.7)	256	0.073 (0.000–0.150)	*p* = 0.218
	Male	1 (0.4)	4 (1.6)	252 (98.1)	257		
Region	Central	0 (0.0)	2 (1.9)	103 (98.1)	105	0.069 (0.000–0.085)	*p* = 0.837
	Eastern	2 (1.5)	3 (2.3)	127 (96.2)	132		
	Northern	1 (0.8)	2 (1.6)	125 (97.7)	128		
	Southern	2 (2.7)	2 (2.7)	71 (94.7)	75		
	Western	0 (0.0)	2 (2.7)	71 (97.3)	73		
Completed recommended vaccinations*	No	0 (0.0)	4 (8.3)	44 (91.7)	48	0.140 (0.041–0.221)	*p* = 0.024
	Yes	5 (1.1)	7 (1.5)	453 (97.4)	465		

* Completed recommended vaccinations indicates that the child received all recommended vaccinations up to age 5 years, or up to the child’s current age if younger than 5 years, as reported by the parent. Cramer’s V is reported with 95% confidence intervals as the omnibus effect-size measure. *p*-values were obtained using Fisher’s exact test because of sparse expected cell counts and are unadjusted for multiple comparisons. CI, confidence interval.

### 3.3 Vaccination attitudes, information sources, and parent-reported practices

Figure 1 summarizes key indicators related to routine childhood vaccination and the HPV module. Awareness of immunization programs in Saudi Arabia was 57.9%, and 61.4% reported being satisfied with vaccination information provided through media. Most parents did not believe vaccination is harmful (71.7%). Receipt and maintenance of an immunization certificate were reported by 59.6 and 60.4%, respectively, and 55.2% stated they would recommend vaccination to others. Within the HPV module, 68.6% reported awareness of the HPV vaccine and 52.4% indicated they would recommend the HPV vaccine for their child (Figure 1).

**Figure 1:**
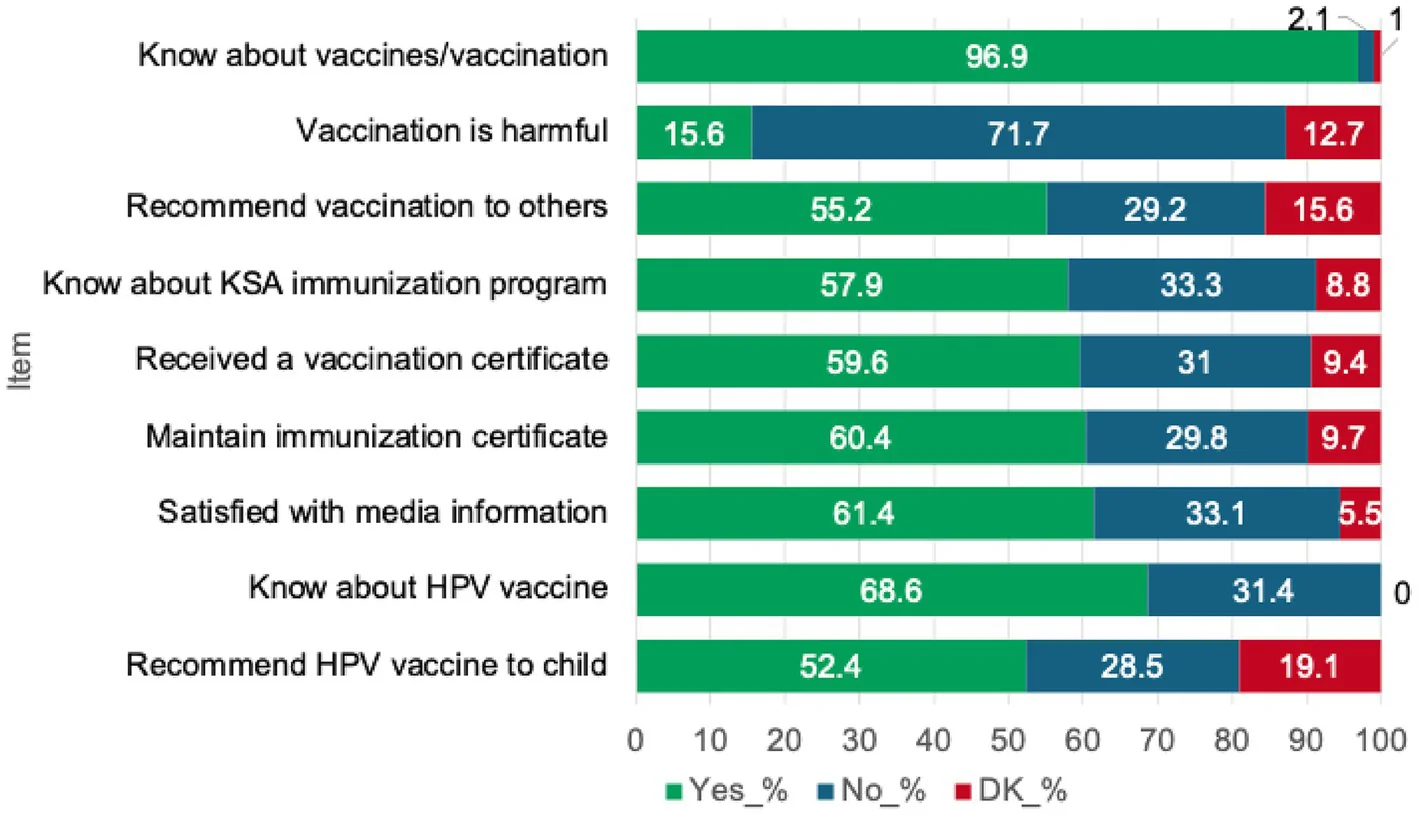
Parental responses to vaccination awareness, perceptions, and related practices (*n* = 513).

Figure 2 shows that improving child immunity was the most frequently selected purpose of childhood vaccination (342/513; 66.7%), followed by helping children grow (287/513; 55.9%) and disease prevention/treatment (265/513; 51.7%). Figure 3 shows that health workers were the most frequently selected source of vaccination information (308/513; 60.0%), followed by friends and relatives (266/513; 51.9%), the Ministry of Health mobile phone service (201/513; 39.2%), and media (194/513; 37.8%). Because respondents could select more than one option, percentages for these multi-response items may exceed 100%. Figure 4 shows high reported uptake of routine childhood vaccination (507/513; 98.8%) and completion of recommended vaccinations up to age 5 years or the child’s current age if younger than 5 years (465/513; 90.6%). Problems during vaccination were reported by 230/513 parents (44.8%), and problems after vaccination by 185/513 (36.1%). A separate item asking whether the child had experienced a parent-reported side effect following vaccination that resulted in a doctor or hospital visit was answered affirmatively by 210/513 parents (40.9%).

**Figure 2:**
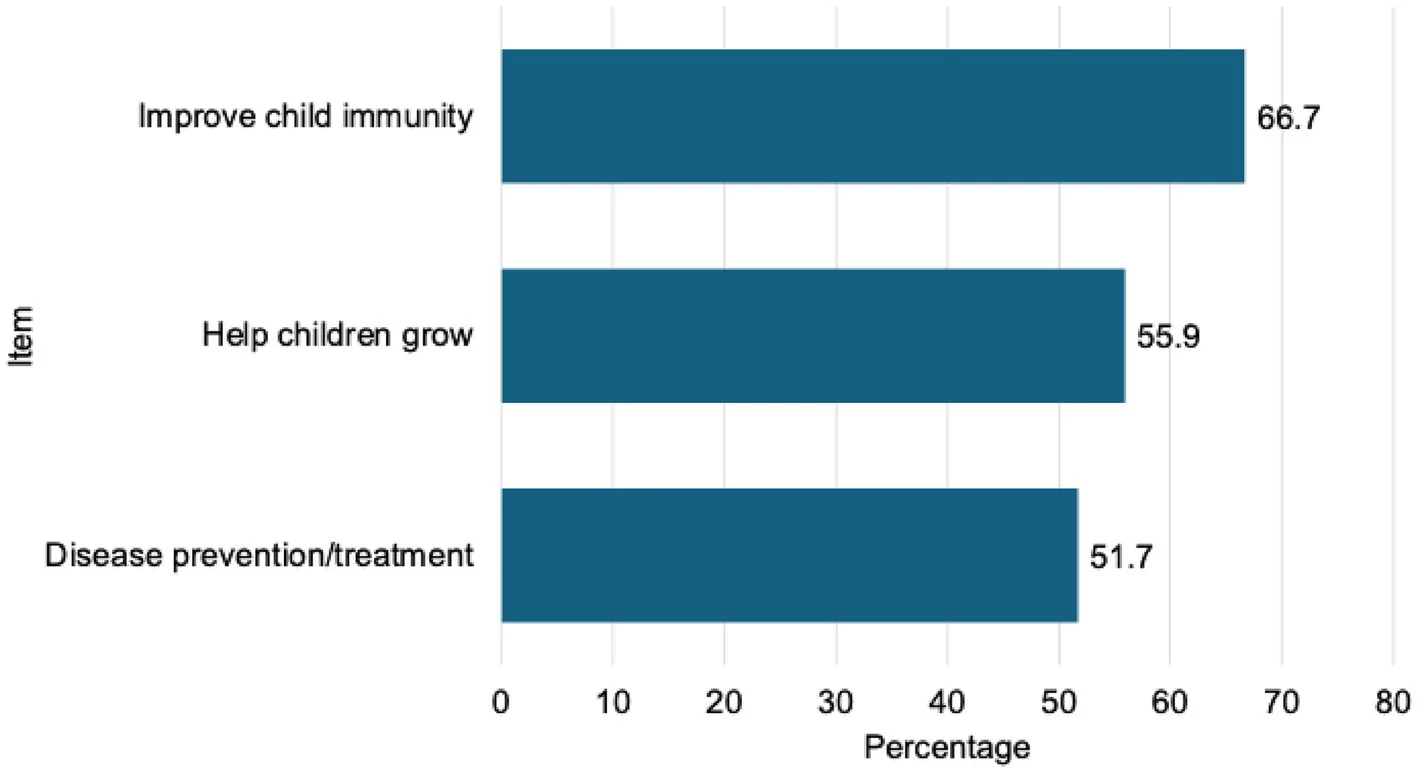
Reported purposes of childhood vaccination among Saudi parents (*n* = 513). Respondents could select more than one option; therefore, percentages may exceed 100%. Responses of “all of the above” were counted toward each corresponding individual vaccination purpose.

**Figure 3:**
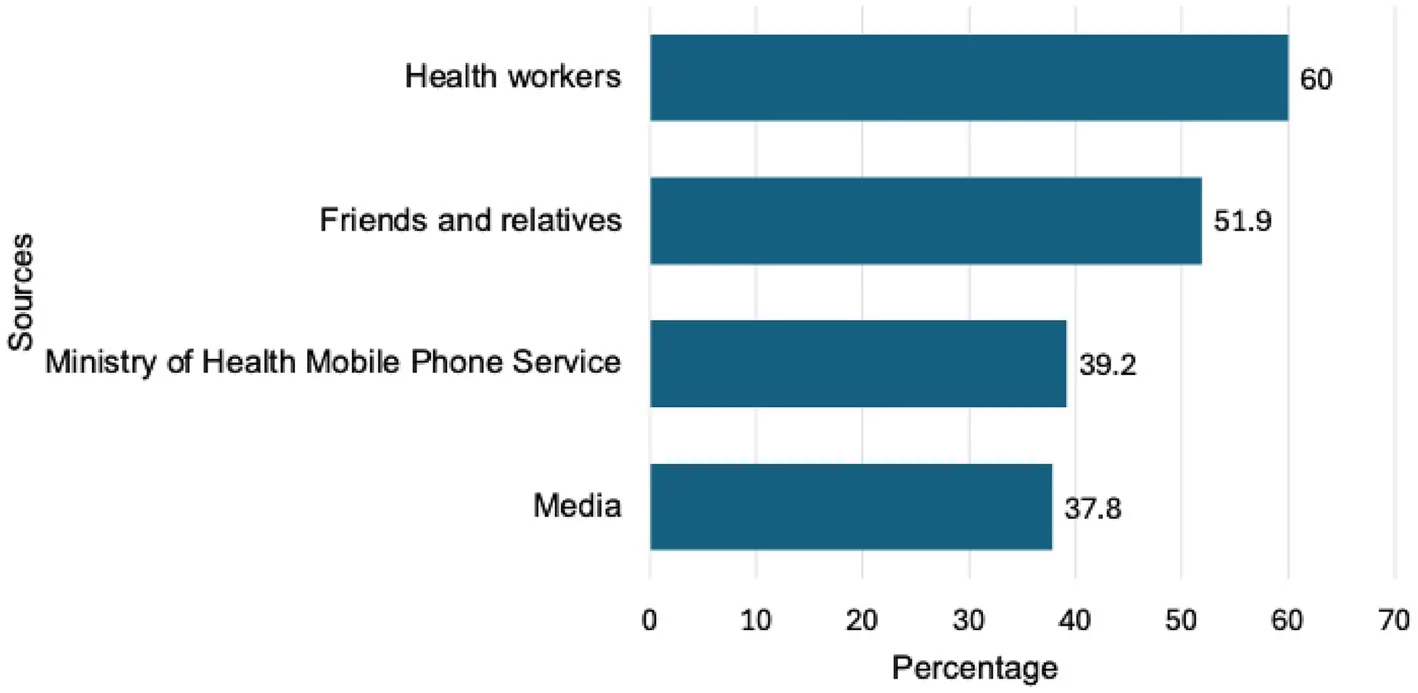
Reported sources of vaccination information among Saudi parents (*n* = 513). Respondents could select more than one option; therefore, percentages may exceed 100%. Responses of “all of the above” were counted toward each corresponding individual information source.

**Figure 4:**
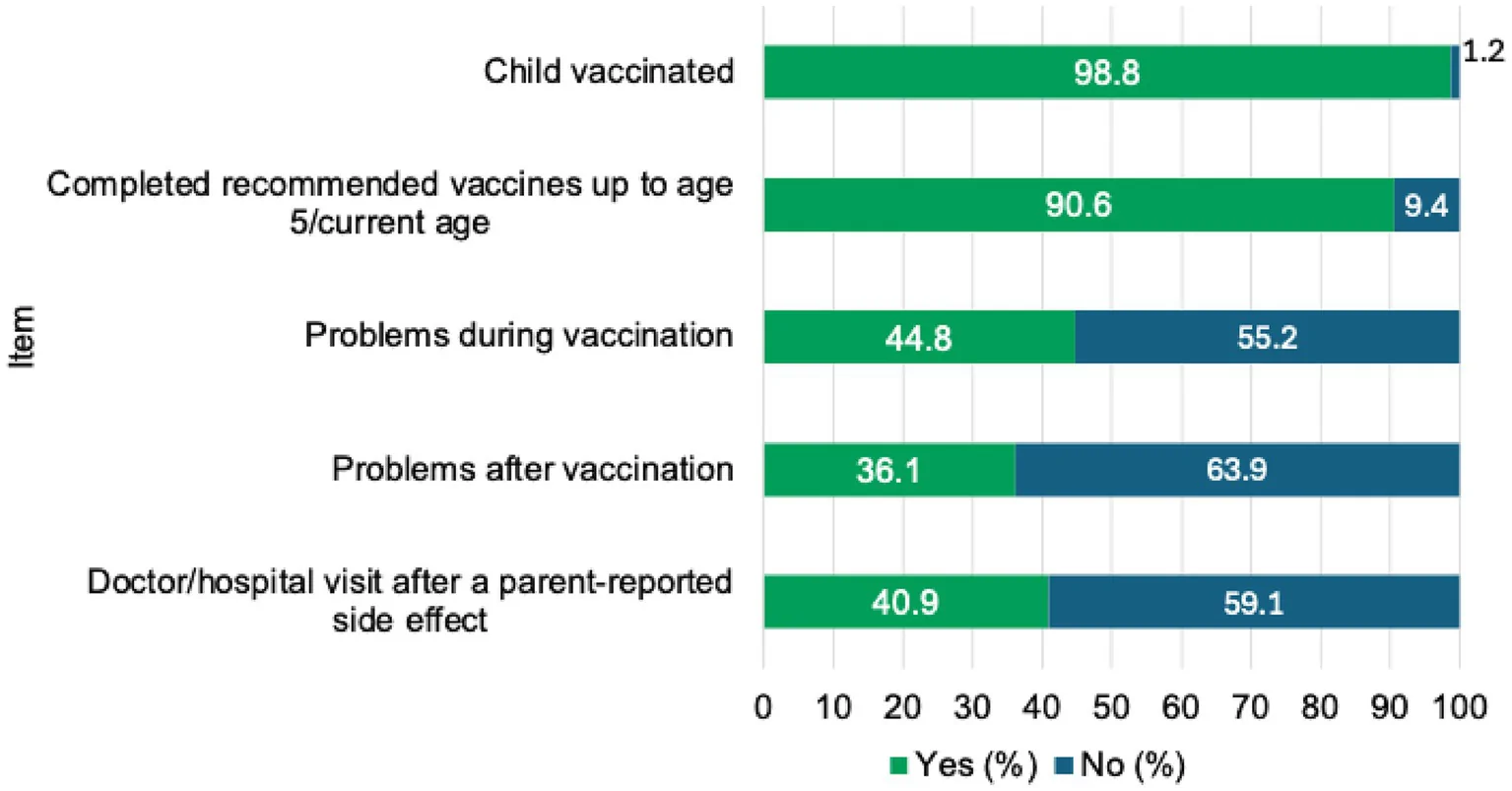
Parent-reported vaccination practices and experiences among Saudi parents (*n* = 513). “Problems during vaccination,” “problems after vaccination,” and “side effect resulting in a doctor/hospital visit” reflect parent-reported questionnaire responses and were not clinically verified. The doctor/hospital-visit item was a separate question administered to all respondents and was not conditional on reporting a problem after vaccination. Percentages are based on the full sample (*n* = 513).

### 3.4 HPV vaccine awareness by participant characteristics

Overall, 352/513 parents (68.6%) reported awareness of the HPV vaccine. In bivariate analyses, females had higher odds of HPV vaccine awareness than males (crude OR = 1.69; 95% CI: 1.16–2.46). Compared with parents in the Southern region, higher odds of awareness were observed in the Central region (OR = 2.78; 95% CI: 1.50–5.13), Eastern region (OR = 5.73; 95% CI: 3.03–10.81), Northern region (OR = 3.01; 95% CI: 1.67–5.45), and Western region (OR = 2.60; 95% CI: 1.33–5.07). Compared with parents who selected “I do not know” regarding vaccine harmfulness, those who responded “No” had higher odds of HPV vaccine awareness (OR = 1.71; 95% CI: 1.00–2.92), as did those who responded “Yes” (OR = 3.80; 95% CI: 1.78–8.09). Parents reporting problems during vaccination also had higher odds of HPV vaccine awareness (OR = 3.39; 95% CI: 2.25–5.12). Age group, educational level, and monthly household income were not significantly associated with HPV vaccine awareness in the bivariate analyses (Table 3).

**Table 3:** Crude associations between factors included in the primary multivariable model and HPV vaccine awareness (*n* = 513).

Factor	Category	No, *n* (%)	Yes, *n* (%)	Total (*n*)	Crude OR (95% CI)	*p*-value
Age (years)	18–35	47 (35.9)	84 (64.1)	131	Reference	0.433
	36–55	91 (30.0)	212 (70.0)	303	1.30 (0.85–2.01)	
	56 years or older	23 (29.1)	56 (70.9)	79	1.36 (0.75–2.49)	
Gender	Male	95 (37.0)	162 (63.0)	257	Reference	0.008
	Female	66 (25.8)	190 (74.2)	256	1.69 (1.16–2.46)	
Region	Southern	42 (56.0)	33 (44.0)	75	Reference	<0.001
	Central	33 (31.4)	72 (68.6)	105	2.78 (1.50–5.13)	
	Eastern	24 (18.2)	108 (81.8)	132	5.73 (3.03–10.81)	
	Northern	38 (29.7)	90 (70.3)	128	3.01 (1.67–5.45)	
	Western	24 (32.9)	49 (67.1)	73	2.60 (1.33–5.07)	
Educational level	High school or less	30 (38.5)	48 (61.5)	78	Reference	0.339
	Bachelor’s degree	103 (30.3)	237 (69.7)	340	1.44 (0.86–2.40)	
	Postgraduate	28 (29.5)	67 (70.5)	95	1.50 (0.79–2.82)	
Monthly household income (SAR)	≤5,000	15 (38.5)	24 (61.5)	39	Reference	0.340
	5,001–10,000	57 (29.7)	135 (70.3)	192	1.48 (0.72–3.03)	
	10,001–20,000	72 (29.9)	169 (70.1)	241	1.47 (0.73–2.96)	
	≥20,000	17 (41.5)	24 (58.5)	41	0.88 (0.36–2.16)	
Perceived vaccination harmfulness	I do not know	29 (44.6)	36 (55.4)	65	Reference	0.002
	No	118 (32.1)	250 (67.9)	368	1.71 (1.00–2.92)	
	Yes	14 (17.5)	66 (82.5)	80	3.80 (1.78–8.09)	
Problems during vaccination	No	120 (42.4)	163 (57.6)	283	Reference	<0.001
	Yes	41 (17.8)	189 (82.2)	230	3.39 (2.25–5.12)	

ORs are crude (unadjusted) odds ratios comparing the odds of reporting HPV vaccine awareness with the stated reference category. Omnibus *p*-values were obtained using Pearson’s chi-square test; Yates’ continuity correction was applied to 2 × 2 tables. All expected cell counts exceeded 5. *p*-values are unadjusted for multiple comparisons. OR, odds ratio; CI, confidence interval.

### 3.5 Factors associated with HPV vaccine awareness

In the revised multivariable model (Table 4), female participants had higher adjusted odds of HPV vaccine awareness than male participants (aOR = 1.87; 95% CI: 1.23–2.86; *p* = 0.004). Compared with the Southern region, higher adjusted odds of awareness were observed in the Central region (aOR = 2.08; 95% CI: 1.04–4.15; *p* = 0.037), Eastern region (aOR = 3.38; 95% CI: 1.68–6.81; *p* < 0.001), Northern region (aOR = 2.02; 95% CI: 1.05–3.91; *p* = 0.036), and Western region (aOR = 2.53; 95% CI: 1.24–5.14; *p* = 0.011).

**Table 4:** Factors associated with HPV vaccine awareness among parents (binary logistic regression; *n* = 513).

Factor	Category	Adjusted OR	95% CI	*p*-value
Age (ref: 18–35 years)	36–55 years	0.97	0.60–1.56	0.887
	56 years or older	0.91	0.45–1.81	0.782
Gender (ref: male)	Female	1.87	1.23–2.86	0.004
Region (ref: southern)	Central	2.08	1.04–4.15	0.037
	Eastern	3.38	1.68–6.81	<0.001
	Northern	2.02	1.05–3.91	0.036
	Western	2.53	1.24–5.14	0.011
Education (ref: high school or less)	Bachelor’s degree	1.47	0.83–2.63	0.188
	Postgraduate	2.09	0.97–4.50	0.060
Monthly household income (ref: ≤SAR 5,000)	SAR 5,001–10,000	1.10	0.50–2.41	0.819
	SAR 10,001–20,000	1.08	0.49–2.39	0.841
	≥SAR 20,000	0.90	0.31–2.60	0.852
Perceived vaccine harmfulness (ref: I do not know)	No	2.10	1.16–3.79	0.014
	Yes	3.44	1.51–7.85	0.003
Problems during vaccination (ref: no)	Yes	2.82	1.76–4.51	<0.001

Reference categories are indicated in the factor labels. aOR, adjusted odds ratio; CI, confidence interval.

Compared with parents who selected “I do not know” when asked whether vaccination is harmful, higher adjusted odds of HPV vaccine awareness were observed among those who responded No (aOR = 2.10; 95% CI: 1.16–3.79; *p* = 0.014) and those who responded Yes (aOR = 3.44; 95% CI: 1.51–7.85; *p* = 0.003). Parents who reported problems during vaccination also had higher adjusted odds of HPV vaccine awareness than those who did not (aOR = 2.82; 95% CI: 1.76–4.51; *p* < 0.001). Age group, educational level, and monthly household income were not statistically significantly associated with HPV vaccine awareness after adjustment.

In the sensitivity analysis additionally incorporating occupation and vaccination-information-source indicators, the direction and magnitude of the principal associations were materially unchanged.

### 3.6 Factors associated with HPV vaccine recommendation intention

In the exploratory multinomial logistic regression, with “Yes” as the reference outcome for HPV vaccine recommendation intention, several factors were associated with the relative likelihood of responding “No” or “I do not know” (Table 5). Compared with parents aged 18–35 years, those aged 36–55 years (aRRR = 2.44; 95% CI: 1.32–4.50; *p* = 0.004) and those aged 56 years or older (aRRR = 2.41; 95% CI: 1.12–5.18; *p* = 0.024) had a higher relative likelihood of responding “No” rather than “Yes.” Compared with the Southern region, the relative likelihood of responding “No” rather than “Yes” was higher in the Central (aRRR = 7.51; 95% CI: 2.34–24.11; *p* < 0.001), Eastern (aRRR = 4.78; 95% CI: 1.54–14.77; *p* = 0.007), and Northern (aRRR = 8.03; 95% CI: 2.61–24.72; *p* < 0.001) regions. Postgraduate education was associated with a lower relative likelihood of responding “No” rather than “Yes” compared with high school education or less (aRRR = 0.34; 95% CI: 0.14–0.81; *p* = 0.014).

**Table 5:** Factors associated with HPV vaccine recommendation intention among parents (multinomial logistic regression; *n* = 513).

Factor	Category	No vs Yes aRRR (95% CI)	*p*-value	I do not know vs Yes aRRR (95% CI)	*p*-value
Age (ref: 18–35 years)	36–55 years	2.44 (1.32–4.50)	0.004	0.68 (0.36–1.31)	0.251
	56 years or older	2.41 (1.12–5.18)	0.024	0.30 (0.10–0.89)	0.030
Gender (ref: male)	Female	0.97 (0.62–1.50)	0.880	0.55 (0.29–1.02)	0.058
Region (ref: southern)	Central	7.51 (2.34–24.11)	<0.001	0.74 (0.30–1.82)	0.510
	Eastern	4.78 (1.54–14.77)	0.007	0.30 (0.11–0.83)	0.020
	Northern	8.03 (2.61–24.72)	<0.001	0.49 (0.20–1.19)	0.116
	Western	3.42 (0.96–12.11)	0.057	0.86 (0.35–2.11)	0.738
Education (ref: high school or less)	Bachelor’s degree	0.68 (0.36–1.30)	0.247	1.27 (0.55–2.96)	0.574
	Postgraduate	0.34 (0.14–0.81)	0.014	1.09 (0.36–3.28)	0.882
Monthly household income (ref: ≤SAR 5,000)	SAR 5,001–10,000	2.21 (0.81–6.05)	0.122	0.68 (0.23–2.02)	0.485
	SAR 10,001–20,000	1.57 (0.57–4.31)	0.382	0.77 (0.25–2.32)	0.642
	≥SAR 20,000	1.14 (0.27–4.78)	0.859	1.87 (0.45–7.74)	0.389
Perceived vaccine harmfulness (ref: I do not know)	No	0.54 (0.28–1.03)	0.060	1.19 (0.48–2.92)	0.705
	Yes	0.38 (0.17–0.83)	0.015	0.24 (0.05–1.14)	0.073
HPV vaccine awareness (ref: no)	Yes	0.92 (0.54–1.56)	0.755	0.07 (0.04–0.13)	<0.001

“Yes” was the reference category for the dependent variable. Reference categories for the independent variables are indicated in the factor labels. aRRR, adjusted relative risk ratio; CI, confidence interval.

For the “I do not know” versus “Yes” comparison, parents aged 56 years or older had a lower relative likelihood of being undecided than those aged 18–35 years (aRRR = 0.30; 95% CI: 0.10–0.89; *p* = 0.030). Participants in the Eastern region also had a lower relative likelihood of responding “I do not know” rather than “Yes” compared with those in the Southern region (aRRR = 0.30; 95% CI: 0.11–0.83; *p* = 0.020). HPV vaccine awareness showed the strongest association with indecision: parents who were aware of the HPV vaccine had substantially lower relative likelihood of responding “I do not know” rather than “Yes” compared with parents who were not aware (aRRR = 0.07; 95% CI: 0.04–0.13; *p* < 0.001).

## 4 Discussion

The main finding of this study is that high reported uptake of routine childhood vaccination can coexist with lower self-reported awareness of, and recommendation intention toward, the HPV vaccine. In our sample, routine vaccination uptake and reported completion of recommended vaccinations were high, whereas HPV vaccine awareness and intention to recommend the HPV vaccine were lower, with a notable proportion of parents selecting “I do not know” for recommendation intention. These findings indicate that high uptake of routine childhood vaccination does not necessarily correspond to similarly high HPV vaccine awareness or recommendation intention. Health workers were an important source of vaccination information, although parents also reported using other information sources. Parent-reported post-vaccination concerns were common, but these reports were not clinically verified and should be interpreted as reported experiences rather than confirmed adverse events.

The earlier Hail study provides a useful baseline for interpretation. It was interviewer administered and reported high completion up to age 5 years and strong encouragement of other parents, while information sources were presented as items respondents could cite in combination rather than as single source patterns (3). In our online survey, graded response options made ‘I do not know’ responses visible, and information sources were summarized as individual multi-select responses. Differences in survey administration and response handling should therefore be considered when comparing percentages with the earlier study. This measurement difference is important in a setting where vaccination attitudes are increasingly shaped by a mixed information environment, including online content that can amplify both reassurance and concern (5, 17).

Self-reported general vaccine awareness was higher in our national online sample (96.9%) than in the earlier Hail survey (78.9%) (3). This difference may reflect differences in geography, sampling frame, and survey mode (interviewer administered versus online) rather than a true change over time. Recent Saudi parent surveys similarly report generally favorable views toward routine childhood vaccination while also documenting misconceptions and subgroup variation in beliefs, including concerns that circulate online (18, 19). A Saudi systematic review and a recent narrative review indicate that childhood vaccine hesitancy remains present across Saudi studies, with concerns commonly linked to safety, trust, and information sources (20, 21). In Riyadh, most parents reported neutral attitudes toward childhood vaccination, suggesting that uncertainty can persist even when routine programs are well established (22). Global coverage trend analyses likewise show that sustaining gains depends on both strong delivery systems and sustained public confidence (23).

Although reported routine vaccination practices were strong in our sample, responses to individual awareness and perception items were more variable. Most parents reported completion of recommended vaccinations and did not perceive vaccination as harmful, whereas responses concerning awareness of the national immunization program, satisfaction with vaccination information in the media, and willingness to recommend vaccination were less uniform. Saudi studies of routine childhood immunization have similarly reported generally favorable parental attitudes while documenting variation in vaccine-related beliefs, safety concerns, and information sources (8, 24). More broadly, Immunization Agenda 2030 emphasizes the importance of sustaining vaccination demand and ensuring that immunization programs reach all populations equitably (2). In our study, parents reporting completed recommended vaccinations were more likely to respond affirmatively to the general vaccine-awareness item. This association should be interpreted at the item level and does not establish greater vaccine knowledge, confidence, or lower vaccine hesitancy among these parents.

Parents’ reported purposes for childhood vaccination help clarify how families in our sample make sense of routine immunization. Most responses emphasized immediate, general protection, while longer term outcomes such as cancer prevention and broader community protection were less prominent in the way parents described vaccine purpose. This matters for the HPV module because the vaccine is most persuasive when it is understood as cancer prevention, yet it can be evaluated through additional social and stigma related lenses that do not typically apply to early childhood vaccines (25). Evidence from message framing studies also suggests that how HPV vaccination is presented can influence parental willingness, and that cancer prevention framing can be helpful for parents who are undecided (26, 27).

Health workers were the leading information source in our sample, while parents also reported using mass media, friends and relatives, and the Ministry of Health mobile reminder service. Broader literature has shown that online and social-media environments can influence vaccine perceptions and may expose parents to both accurate and misleading information (4, 5). Repeated online narratives, even when unfounded, can shape risk perception and trust and influence how parents interpret post-vaccination symptoms or approach less familiar vaccines. However, the present study did not specifically assess social-media use or misinformation exposure, and these mechanisms should not be inferred from our findings. Within the HPV module, this is especially relevant: provider communication is consistently associated with higher uptake, and guidance for health workers emphasizes brief, clear recommendations supported by simple explanations of benefits, expected reactions, and eligibility (28, 29). Experience from HPV introduction efforts in multiple countries similarly highlights the value of targeted communication and community engagement led by health workers when parents have questions or remain undecided about HPV vaccination (30).

HPV vaccine awareness did not vary meaningfully by age group in our sample, which differs from some Saudi findings where poorer awareness has been associated with older age (31) and from wider MENA evidence showing that age related patterns are inconsistent across settings and study populations (32). By contrast, the gender pattern in our data was more consistent with the broader literature: awareness was higher among females than males, aligning with Saudi evidence reporting lower awareness among fathers and with regional syntheses noting lower HPV awareness among men (31, 32). We also observed clear regional variation within Saudi Arabia, with the lowest HPV vaccine awareness in the Southern region and higher awareness in the other regions. This finding is consistent with previous Saudi evidence showing greater HPV vaccine hesitancy among parents residing in the Southern region (15). A study conducted in Jazan also reported low overall HPV knowledge despite a predominantly university-educated sample, suggesting that regional differences may partly reflect gaps in HPV-specific knowledge and awareness (33). Potential explanations raised in previous Saudi studies include differences in healthcare-provider recommendation, access to vaccine-related information, and local contextual beliefs. However, these factors were not directly assessed in the present study and therefore cannot be identified as causes of the observed regional difference (34).

In multivariable analysis, HPV vaccine awareness was higher among females and among parents living outside the Southern region. Awareness was also higher among parents who expressed a definite view on whether vaccination is harmful, compared with those selecting “I do not know,” and among those reporting problems during vaccination. The association between responses to the vaccine-harmfulness item and HPV vaccine awareness should be interpreted strictly as an association between these individual questionnaire responses; it does not indicate greater vaccine knowledge, confidence, acceptance, or exposure to vaccine-related information. Similarly, the association between parent-reported problems during vaccination and HPV vaccine awareness should be interpreted cautiously because these experiences were self-reported, were not clinically verified, and the cross-sectional design does not establish whether they preceded or influenced HPV vaccine awareness. Educational level and household income were not independently associated with HPV vaccine awareness after adjustment.

The exploratory multinomial analysis of HPV vaccine recommendation intention showed that the factors associated with recommendation intention were not identical to those associated with HPV vaccine awareness. Older age and residence in selected regions were associated with a higher relative likelihood of responding “No” rather than “Yes,” whereas postgraduate education was associated with a lower relative likelihood of responding “No” rather than “Yes.” HPV vaccine awareness was strongly associated with a lower relative likelihood of responding “I do not know” rather than “Yes.” This latter association indicates that awareness and decisional uncertainty were related in this sample, but the cross-sectional design does not establish directionality. Similarly, selecting “I do not know” in response to the HPV vaccine recommendation item reflects an undecided response to that specific question and should not be interpreted as a validated measure of vaccine hesitancy. The sizeable proportion of parents who did not express a definite recommendation intention may nevertheless represent an important group for further investigation and for clear, evidence-based HPV vaccine communication, consistent with guidance and evidence supporting structured clinician communication to improve HPV vaccination uptake (29, 35). The multinomial findings should also be interpreted cautiously, particularly where confidence intervals were wide, and are best regarded as exploratory associations rather than determinants of recommendation intention.

A key strength of this study is that it extends a prior Saudi immunization study using an adapted instrument in a broader, multi region online sample, while also incorporating HPV-specific items assessing awareness and recommendation intention. Several limitations should be considered. The online non-probability convenience design limits generalizability and may overrepresent parents with greater internet access, those who are more digitally engaged, those reached through the investigators’ networks and those with higher educational attainment, potentially affecting the observed distributions of vaccination awareness, perceptions, and reported practices. Recruitment through investigators’ networks may also have contributed to uneven representation across regions; therefore, regional differences should be interpreted cautiously. All measures were self-reported, and parent-reported post vaccination events were not clinically verified, so recall and attribution bias are possible. The cross-sectional design limits causal inference, and the observed associations should not be interpreted as evidence of temporal or causal relationships. Despite adjustment for the measured sociodemographic, perception-related, and vaccination-experience variables and the additional sensitivity analysis, residual confounding from unmeasured or imperfectly measured factors cannot be excluded. Finally, we did not use validated scales to measure vaccine knowledge, confidence, hesitancy, acceptance, or HPV-specific knowledge; therefore, these constructs should not be inferred from responses to individual awareness or perception items. Despite these constraints, the findings help identify where differences in awareness, perceptions, and recommendation intention are most apparent and can inform future research and communication strategies.

## 5 Conclusion

In this convenience sample of Saudi parents, reported uptake of routine childhood vaccination was high, whereas HPV vaccine awareness and intention to recommend HPV vaccination were lower. HPV vaccine awareness varied by gender and region and was associated with responses to the vaccine-harmfulness item and with parent-reported problems during vaccination. In the exploratory analysis of HPV vaccine recommendation intention, the pattern of associated factors differed from that observed for HPV vaccine awareness, and a substantial proportion of parents selected “I do not know.” These findings highlight the need for further research using representative sampling and validated HPV-specific measures to better understand the factors underlying HPV vaccine awareness and recommendation intention. Clear, evidence-based communication about HPV vaccination may also be particularly relevant for parents who remain undecided.

## Data Availability

The raw data supporting the conclusions of this article will be made available by the authors, without undue reservation.
