# Supplementary material for: Parental perceptions, information sources, and practices related to childhood immunization and HPV vaccine awareness in Saudi Arabia: an observational study
Source: Front Public Health. 2026 Oct 22;14:1992535. doi: 10.3389/fpubh.2026.1992535 (PMC13736608; doi:10.3389/fpubh.2026.1992535)
Supplement: Supplementary file 1 [file Supplementary_file_1.docx]

**Supplementary Material
Questionnaire - English Version**

*Parental Perceptions, Information Sources, and Practices Related to Childhood Immunization and HPV Vaccine Awareness in Saudi Arabia: An Observational Study*

**Section 1. Participant Characteristics**

**1. Gender**

(a) Male

(b) Female

**2. Region**

(a) Southern

(b) Northern

(c) Eastern

(d) Western

(e) Central

**3. Age (years)**

(a) 18–35

(b) 36–55

(c) 56 years or older

**4. Education level**

(a) No formal education

(b) Primary school

(c) Middle school

(d) High school

(e) Bachelor’s degree

(f) Postgraduate

**5. Occupation sector**

(a) Military

(b) Health services

(c) Agriculture

(d) Education (teaching)

(e) Tourism

(f) Industry

(g) Financial services

(h) Free trade

(i) Legal

(j) Student

(k) Retired

(l) Unemployed

**6. Number of children younger than 5 years**

(a) 1

(b) 2

(c) 3

(d) 4

(e) Other (please specify)

**7. Monthly household income (SAR)**

(a) ≤5,000

(b) 5,001–10,000

(c) 10,001–20,000

(d) ≥20,000

**Section 2. Vaccination Awareness and Perceptions**

**1. Do you know about vaccines?**

(a) Yes

(b) No

(c) I don’t know

**2. Is vaccination harmful?**

(a) Yes

(b) No

(c) I don’t know

**3. What is the purpose of vaccination?** *(You may select more than one answer.)*

(a) Preventing/treating disease

(b) Improving child immunity

(c) Helping children grow

(d) All of the above (all listed purposes)

**4. Would you recommend vaccination to others?**

(a) Yes

(b) No

(c) I don’t know

**5. How do you obtain information about vaccination?** *(You may select more than one answer.)*

(a) Mass media

(b) Health workers

(c) Friends and relatives

(d) Ministry of Health mobile reminder service

(e) All of the above (all listed sources)

**6. Are you aware of any immunization program in Saudi Arabia?**

(a) Yes

(b) No

(c) I don’t know

**7. Have you received an immunization certificate for your children?**

(a) Yes

(b) No

(c) I don’t know

**8. Do you keep (maintain) the immunization certificate?**

(a) Yes

(b) No

(c) I don’t know

**9. Are you satisfied with the information about vaccination provided through the media?**

(a) Yes

(b) No

(c) I don’t know

**10. Are you aware of the human papillomavirus (HPV) vaccine?**

(a) Yes

(b) No

**11. Would you recommend the human papillomavirus (HPV) vaccine for your child?**

(a) Yes

(b) No

(c) I don’t know

**Section 3. Parent-Reported Vaccination Practices and Experiences**

**1. Has your child been vaccinated?**

(a) Yes

(b) No

**2. Has your child experienced any problems during vaccination?**

(a) Yes

(b) No

**3. Has your child experienced any problems after vaccination?**

(a) Yes

(b) No

**4. Has your child experienced a side effect following vaccination that required a doctor or hospital visit**

(a) Yes

(b) No

**5. Has your child completed all required vaccines up to age 5/preschool (or up to their current age if younger than 5 years)?**

(a) Yes

(b) No

*Thank you*
